# Dietary acid load and risk of diminished ovarian reserve: a case-control study

**DOI:** 10.1186/s12958-024-01238-2

**Published:** 2024-06-04

**Authors:** Rahele Ziaei, Abed Ghavami, Hatav Ghasemi-Tehrani, Minoo Movahedi, Maryam Hashemi, Maryam Hajhashemi, Mahshid Elyasi, Mahdi Vajdi, Maryam Kalatehjari

**Affiliations:** 1 Department of Community Nutrition, School of Nutrition and Food Science, Isfahan University of Medical Sciences, Isfahan, Iran; 2 Fertility department, School of Medicine, Isfahan University of Medical Sciences, Isfahan, Iran; 3 Department of Obstetrics & Gynecology, School of Medicine, Isfahan University of Medical Sciences, Isfahan, Iran; 4 Department of Clinical Nutrition, School of Nutrition and Food Science, Shiraz University of Medical Sciences, Shiraz, Iran; 5 Student Research Committee, Department of Community Nutrition, School of Nutrition and Food Science, Isfahan, Iran; 6 Reproductive Sciences and Sexual Health Research Center, Isfahan University of Medical Sciences, Isfahan, Iran

**Keywords:** Dietary acid load, Diminished ovarian reserve, Antimullerian hormone, Potential renal acid load, Antral follicle count

## Abstract

**Background:**

The epidemiologic evidence on the association between acid load potential of diet and the risk of diminished ovarian reserve (DOR) is scarce. We aim to explore the possible relationship between dietary acid load (DAL), markers of ovarian reserve and DOR risk in a case-control study.

**Methods:**

370 women (120 women with DOR and 250 women with normal ovarian reserve as controls), matched by age and BMI, were recruited. Dietary intake was obtained using a validated 80-item semi-quantitative food frequency questionnaire (FFQ). The DAL scores including the potential renal acid load (PRAL) and net endogenous acid production (NEAP) were calculated based on nutrients intake. NEAP and PRAL scores were categorized by quartiles based on the distribution of controls. Antral follicle count (AFC), serum antimullerian hormone (AMH) and anthropometric indices were measured. Logistic regression models were used to estimate multivariable odds ratio (OR) of DOR across quartiles of NEAP and PRAL scores.

**Results:**

Following increase in PRAL and NEAP scores, serum AMH significantly decreased in women with DOR. Also, AFC count had a significant decrease following increase in PRAL score (*P* = 0.045). After adjustment for multiple confounding variables, participants in the top quartile of PRAL had increased OR for DOR (OR: 1.26; 95%CI: 1.08–1.42, *P* = 0.254).

**Conclusion:**

Diets with high acid-forming potential may negatively affect ovarian reserve in women with DOR. Also, high DAL may increase the risk of DOR. The association between DAL and markers of ovarian reserve should be explored in prospective studies and clinical trials.

## Introduction

Ovarian reserve is determined by the number and quality of remaining oocytes, both decline by age. Diminished ovarian reserve (DOR) is when a woman in reproductive age with regular menses has reduced fecundity or decreased response to ovarian stimulation compared to women with similar age [1]. Antral follicle count (AFC), which is measured using ultrasound and serum antimullerian hormone (AMH) level are best currently available determinants of ovarian reserve [1]. The diagnosis is based on decreased AFC and/or abnormal serum hormones (i.e., elevated FSH and low AMH levels) in a woman with regular menstrual cycles [2]. DOR has been shown to be associated with unfavorable fertility and assisted reproductive technologies (ART) outcomes [3].

Although, the exact etiology of DOR remains idiopathic in most cases, several factors including genetic factors, autoimmune diseases, iatrogenic causes and environmental factors were proposed to cause DOR [2]. The fact that females of the same age have various reproductive potential may indicate the impact of environmental factors on ovarian reserve [4]. Identifying potentially modifiable factors including nutritional factors which could promote ovarian reserve and thus beneficially affect fertility has been the focus of several recent observational studies [5, 6].

Diet is a major contributing factor to acid-base imbalance, as western-style diets which are rich in acidogenic foods (animal products and processed wheat-based products) and low in alkaline foods (fruit and vegetables) are associated with high diet-induced acid load. Endogenous acid production (NEAP) score, which is based on total protein and potassium intake and the potential renal acid load (PRAL) score which is based on intake of protein, phosphorus, potassium, magnesium and calcium are two validated scores to estimate dietary acid load (DAL). Based on recent evidence, high DAL might be a risk factor for cardiovascular diseases and metabolic disorders such as insulin resistance and type 2 diabetes mellitus [7–10].

It has been proposed that oxidative stress followed by metabolic derangements might affect ovarian reserve, as antioxidant compounds were successfully used to improve ovarian reserve [11–13]. Both DOR and high acid-forming potential of diet have been linked to metabolic disorders and increased cardiovascular risk. We hypothesized that there might be an association between DAL and decreased ovarian reserve and lowering acid-forming potential of diet may beneficially affect ovarian reserve in women with DOR. So, we conducted a case-control study to investigate the association between diet-induced acid load, using both PRAL and DAL scores, with markers of ovarian reserve including serum AMH and AFC in women with DOR as well as risk of DOR.

## Methods

For this case control study, 370 women (120 women with DOR and 250 women with normal ovarian reserve as controls) of 18 to 45 years and with body mass index (BMI) between 20 and 35 kg/m^2^ were recruited from infertility centers through purposive sampling. Participants were excluded if they; were current or previous (within the last 3 months) users of oral contraceptive drugs, hormone therapy, weight-loss interventions and multivitamin mineral supplements; had a history of ovarian surgery, chemotherapy or radiotherapy, premature ovarian failure, infertility treatment, endometriosis, endocrine disorders including polycystic ovary syndrome, thyroid disorders, diabetes or impaired glucose tolerance, Cushing’s syndrome, hyperprolactinemia and androgenic disorders, a major chronic disease (e.g., gastrointestinal diseases, cancer, cardiovascular diseases, liver or kidney disorders and mood disorders, all based on patients’ medical records); were following specific diet or physical activity programs; were current smokers or consumed alcohol. Participants with incomplete FFQ, who answered less than 35 items of the FFQ, and those with implausible total energy intake (< 500 and > 3,500 kcal/day) were also excluded. Each woman with DOR was paired with two women with normal ovarian reserve by age and BMI. DOR diagnosis was made by an expert gynecologist, as women with either low AMH level (≤ 0.7 ng/mL) or low AFC (≤ 4 in both ovaries) or both of them were considered to have decreased ovarian reserve [1]. Women with normal ovarian reserve were randomly selected from the same infertility center. The study was conducted in accordance with the Declaration of Helsinki [14]. Participants were provided with an information sheet explaining the study protocol, and consented to participate. The study protocol was approved by the local Ethics Committee of Isfahan University of Medical Sciences (IR.ARI.MUI.REC.1401.297).

### Dietary intake and physical activity measurements

Dietary information was obtained using a validated 80-item semi-quantitative food frequency questionnaire (FFQ) [15]. For each food item, women were asked how often, on average, over the previous year, they had consumed the food. Quantifications of food items were based on commonly used units. Six response categories per food item (never, 2–3 times/ month, 1 time/week, 2–4 times/week, 5–6 times/week, and daily) were considered for each food. Data were transformed to daily intake frequency. Portion sizes consumed from each food item were converted into grams, using standard Iranian household measures [16]. Daily food consumption was computed by multiplying the daily frequency of intake by portion size for each food item. Dietary intakes were then analyzed using the Nutritionist-4 software (First Databank Inc. San Bruno, CA), modified for Iranian foods. To calculate the physical activity, a short form of the International Physical Activity Questionnaire (IPAQ) was used to determine the metabolic equivalent (MET) minute per week [17]. The duration and frequency of physical activity days have multiplied by the activity’s MET value to get the MET minute per week (MET/min/wk). The total weekly exercise minute was then determined by summing the scores.

### Dietary acid load

PRAL and NEAP are two of the most important factors that describe DAL. PRAL is an estimate of the amount of acid produced by the body that is greater than the amount of alkali produced. This number is based on the foods eaten every day. Considerably, meats, eggs, and dairy products are acid-producing foods, while most fruits and vegetables are base-producing foods. By using the Remer and Manz calculation model, we calculated the PRAL of food intake from the 80-item FFQ [15].

[PRAL (mEq/d) = 0.49*protein(g) + 0.037*phosphorus (mg)] - 0.021*potassium (mg) 0.026*magnesium (mg)‐0.0125*calcium (mg)] [16].

As a result of the balance between acid and alkali precursors in the diet, the nonvolatile acid load, also referred to as the NEAP, is calculated [18]. An equation that had previously been validated was utilized to estimate NEAP in this study: [NEAP (mEq/d) = − 10.2 + 54.5 (protein intake [g/d] ÷ potassium intake [mEq/d])] [19].

Due to the fact that different variables are used to calculate DAL in these formulas, and there still is no consistent mechanism to be able to determine which variable has greater credibility, we used both variables in the present research.

### AFC and AMH measurement

Transvaginal ultrasound was performed to determine the total AFC by an infertility gynecologist, which was calculated as the sum of antral follicles measuring 2–10 mm in both ovaries on the third day of an unstimulated menstrual cycle. Serum AMH levels were assessed using ELISA kit (Monobind, California, USA).

### Assessment of other variables

Participants completed a general demographic questionnaire and the Iranian version of international physical activity questionnaire (IPAQ), which is a valid and reliable questionnaire was used to measure and report PA levels as metabolic equivalent hours per day (MET/h/day) [20]. The demographic questionnaire contained questions on age, education, occupation, anthropometric measures, obstetric history (including DOR duration, history of infertility and previous pregnancy), history of chronic diseases, past and present use of contraceptives, dietary supplements, weight-reducing drugs or other drugs and past and present smoking status. Body weight was measured with minimal clothing and without shoes by a digital Seca scale (Saca 831, Hamburg, Germany), to the nearest 0_·_1 kg. Height was measured in a standing position without shoes using a portable stadiometer (Seca, Hamburg, Germany) to the nearest 0.5 cm. Body mass index (BMI) was calculated as weight divided by height square (kg/m^2^). Waist circumference.

(WC) and hip circumference (HC) were measured twice to the nearest 0.1 cm with a tape measure. The lowest rib and iliac crest’s midpoint and the largest circumference around the buttocks were used to calculate WC and HC respectively. The waist to hip ration (WHR) was then computed by dividing the measured WC (cm) by the measured HC (cm). Fat mass (FM) was estimated using Bio-Impedance Analyzer (BIA) (Inbody 770, Inbody Co, Seoul, Korea). Two blood pressures, systolic and diastolic, were taken in the sitting position and after 5 min of rest using an automated digital sphygmomanometer (Microlife Blood Pressure Monitor A100- 30, Berneck, Switzerland).

### Statistical analyses

The statistical analyses were carried out using SPSS (version 21.0, SPSS Inc., Chicago, Illinois, USA). Mean and standard deviation were used to show quantitative data, while frequency (numbers and percentages) was used to show emotional data. The Kolmogorov-Smirnov test was used to check the normality of the quantitative variables. All participants were classified based on their PRAL and NEAP quartiles. Depending on the nature of the data, the independent samples t-test or chi-square test was used to compare variables between cases and controls. The relationships of PRAL and NEAP with the odds of DOR were investigated using multivariable logistic regression that adjusted for multiple covariates in different models. First, the DOR risk was calculated by means the results identified PRAL and NEAP in the crude model. Multiple possible confounding factors have been adjusted in the final regression model, including energy intake, FM and BMI. In this research, the significance levels were considered at P-values < 0.05.

## Results

Table 1. presents sociodemographic characteristics, body composition and anthropometric indices, physical activity and DOR markers between case and control groups. The mean of BMI in women with DOR was higher than women with normal ovarian reserve (29.85 ± 2.49 vs. 28.75 ± 3.45). Our findings showed that women with DOR had higher mean of FM than women in the control group (38.47 ± 7.05 vs. 36.47 ± 8.91; *P* = 0.020). Also, the results of anthropometric indices showed that WC (102.23 ± 35.95 vs. 91.70 ± 12.43; *P* = 0.002 and WHR (109.10 ± 31.59 vs. 106.10 ± 11.57; *P* = 0.003) were significantly higher in women with DOR compared to control group. The DOR duration was (5.59 ± 4.16) among women with DOR. The mean serum levels of AMH (0.56 ± 0.71 vs. 4.11 ± 1.18; *P* < 0.001) and AFC count (2.34 ± 1.19 vs. 9.59 ± 2.24; *P* < 0.001) were significantly lower in women with DOR compared to women in control group.

**Table 1:** Baseline characteristic of participants

Variable		Case (*N* = 120)	Control (*N* = 250)	*P*-value^a^
Age (years)		33.37 ± 3.24	32.91 ± 3.15	0.196
BMI (kg/m^2^)		29.85 ± 2.49	28.75 ± 3.45	0.235
Weight (kg)		80.96 ± 4.78	79.26 ± 8.41	0.487
FM (kg)		38.47 ± 7.05	36.47 ± 8.91	0.020
FFM (kg)		57.99 ± 11.33	60.12 ± 11.97	0.098
WC (cm)		102.23 ± 35.95	91.70 ± 12.43	0.002
HC (cm)		109.10 ± 31.59	106.10 ± 11.57	0.316
WHR		0.90 ± 0.12	0.86 ± 0.08	0.003
SBP (mmHg)		122.18 ± 12.77	123.58 ± 14.03	0.341
DBP (mmHg)		79.41 ± 11.67	81.85 ± 10.48	0.056
Physical activity (MET/h/day)		19.05 ± 4.12	18.98 ± 4.51	0.896
Socioeconomic status (SES) (%)	Low	10 (8.3)	19 (7.6)	0.252
	Middle	50 (41.7)	127 (50.8)	
	High	60 (50)	104 (41.6)	
Education (%)	Illiterate	14 (11.7)	34 (13.6)	< 0.001
	≤ High school/diploma	31(25.8)	121 (48.4)	
	≥ College degree	75 (62.5)	95 [37]	
Occupation (%)	Housewife	82 (68.3)	184 (73.6)	< 0.001
	Employed	26 (21.7)	10 [4]	
	Student	12 [10]	56 (22.4)	
Pervious Pregnancy	Yes	99 (82.5)	203 (81.2)	0.441
	No	21 (17.5)	47 (18.8)	
AFC count		2.34 ± 1.19	9.59 ± 2.24	< 0.001
AMH (ng/ml)		0.56 ± 0.71	4.11 ± 1.18	< 0.001

Quantitative variables are expressed as mean ± SD and qualitative variables expressed as n (%). *Abbreviation*: AFC, antral follicle count; BMI, body mass index; DBP, diastolic blood pressure; DOR duration, diminished or decreased ovarian reserve FFM, fat free mass; FM, fat mass; HC, hip circumference; SBP, systolic blood pressure; WC, waist circumference; WHR, waist to hip ratio

The SES scored was evaluated based on education level of both subjects and the family head, job of both subjects and the family head family size, home status and home type by using self-reported questionnaire. ^a^ p values resulted from independent t-tests for quantitative and Chi-square for qualitative variables between the two groups

The baseline participant characteristics across the quartiles of PRAL and NEAP were reported in Table 2. Our findings demonstrated that following increase in PRAL and NEAP scores, AMH serum levels had a significant decrease in women with DOR. Also, AFC count had a significant decrease following increase in PRAL score (*P* = 0.045). Among body composition indices, FM significantly increased in women with DOR across PRAL and NEAP scores.

**Table 2:** Characteristic of study participants according to quartiles of potential renal acid load (PRAL) and net endogenous acid production (NEAP)

		PRAL					NEAP				
		Q1(33/61)	Q2(28/59)	Q3(32/61)	Q4(27/69)	P	Q1(34/57)	Q2(32/56)	Q3(28/66)	Q4(26/71)	P^a^
**DOR indices**											
AMH	Case	1.13 ± 0.42	0.98 ± 0.22	0.62 ± 0.15	0.34 ± 0.25	0.023	0.97 ± 1.30	0.50 ± 0.22	0.41 ± 0.17	0.28 ± 0.23	0.034
	Control	4.20 ± 1.11	3.95 ± 1.24	4.20 ± 1.20	4.08 ± 1.19	0.602	4.18 ± 1.14	4.00 ± 1.22	4.21 ± 1.20	4.04 ± 1.18	0.701
AFC	Case	2.60 ± 1.34	2.42 ± 1.23	2.43 ± 1.10	1.81 ± 0.96	0.045	2.52 ± 1.39	2.37 ± 1.12	2.35 ± 1.12	2.03 ± 1.19	0.475
	Control	9.36 ± 2.11	9.71 ± 2.31	10.01 ± 2.34	9.31 ± 2.17	0.259	9.42 ± 2.11	9.83 ± 2.44	9.74 ± 2.14	9.39 ± 2.28	0.604
**Anthropometric indices and body composition**											
BW	Case	82.42 ± 3.89	81.64 ± 3.92	82.40 ± 4.26	82.86 ± 4.35	0.729	82.55 ± 3.97	82.00 ± 4.25	82.67 ± 4.62	82.11 ± 3.60	0.902
	Control	78.11 ± 5.23	78.71 ± 4.35	77.75 ± 5.54	78.11 ± 4.51	0.761	77.84 ± 5.43	78.72 ± 4.57	78.08 ± 4.95	78.05 ± 4.74	0.800
BMI	Case	29.87 ± 2.16	29.98 ± 3.31	29.84 ± 2.16	29.67 ± 2.41	0.978	29.83 ± 2.38	29.86 ± 2.98	30.01 ± 2.32	29.68 ± 2.29	0.972
	Control	27.47 ± 3.57	27.94 ± 3.64	27.43 ± 3.22	28.10 ± 3.35	0.613	27.69 ± 3.52	27.74 ± 3.73	27.52 ± 3.61	28.00 ± 3.03	0.874
WC	Case	109.54 ± 36.38	91.82 ± 28.64	105.56 ± 38.17	100.14 ± 38.65	0.255	107.20 ± 36.23	96.21 ± 32.17	99.71 ± 35.56	105.23 ± 40.85	0.589
	Control	89.59 ± 13.29	92.27 ± 11.38	93.19 ± 13.80	91.76 ± 11.20	0.431	90.82 ± 12.49	91.73 ± 13.21	92.10 ± 12.60	92.01 ± 11.82	0.941
WHR	Case	0.93 ± 0.14	0.87 ± 0.12	0.89 ± 0.11	0.90 ± 0.10	0.274	0.91 ± 0.10	0.89 ± 0.16	0.88 ± 0.09	0.91 ± 0.11	0.675
	Control	0.85 ± 0.08	0.89 ± 0.08	0.86 ± 0.09	0.85 ± 0.07	0.033	0.85 ± 0.07	0.86 ± 0.08	0.87 ± 0.09	0.85 ± 0.07	0.382
FM	Case	35.58 ± 7.20	36.73 ± 9.26	38.32 ± 5.71	43.47 ± 5.76	0.038	32.10 ± 7.06	39.33 ± 9.17	41.62 ± 4.00	75.11 ± 6.46	0.055
	Control	34.74 ± 8.12	36.09 ± 7.28	36.13 ± 9.10	36.87 ± 10.41	0.943	36.03 ± 8.42	35.71 ± 7.98	36.76 ± 8.44	37.17 ± 10.41	0.792
FFM	Case	60.18 ± 11.77	57.82 ± 11.85	57.45 ± 11.74	56.14 ± 9.84	0.571	60.62 ± 12.60	58.32 ± 10.95	55.53 ± 11.61	58.80 ± 9.46	0.328
	Control	59.92 ± 12.87	60.15 ± 12.02	60.36 ± 11.97	60.06 ± 11.36	0.998	60.22 ± 12.84	60.79 ± 12.05	59.92 ± 12.33	59.70 ± 11.05	0.964
**Blood pressure parameters**											
SBP	Case	123.36 ± 14.73	122.36 ± 14.73	122.64 ± 12.00	121.11 ± 11.78	0.897	123.50 ± 14.53	124.31 ± 12.82	119.75 ± 10.10	120.46 ± 12.85	0.438
	Control	123.03 ± 13.64	126.86 ± 15.86	119.83 ± 12.34	124.56 ± 13.60	0.354	123.68 ± 12.87	122.94 ± 16.67	123.40 ± 13.95	124.15 ± 12.98	0.970
DBP	Case	81.15 ± 12.83	80.78 ± 11.84	77.50 ± 12.22	78.14 ± 9.23	0.519	80.88 ± 12.94	81.18 ± 11.07	76.14 ± 11.76	78.84 ± 10.34	0.319
	Control	81.29 ± 10.86	82.96 ± 12.49	80.00 ± 11.03	83.04 ± 8.96	0.565	82.08 ± 10.50	79.82 ± 12.61	82.57 ± 11.57	82.60 ± 8.73	0.455

^a^ANOVA test used for continuous variables; Chi-square test used for categorical variables

Crude and multivariable-adjusted odds ratios (ORs) for the association between DAL based on PRAL and NEAP scores are outlined in Table 3. In the crude model, no significant relationship was found between DAL based on PRAL (OR: 1.28; 95%CI: 0.88–1.76, *P* = 0.380) and NEAP (OR: 1.95; 95%CI: 0.52–2.75, *P* = 0.078) with DOR. This relationship remained non-significant after adjustment for potential confounders including energy intake and physical activity [(PRAL (OR: 1.75; 95%CI: 0.39–2.44, *P* = 0.258) and NEAP (OR: 1.95; 95%CI: 0.52–2.75, *P* = 0.045)]. After further controlling for FM, weight and BMI, we found that patients in the top quartile of PRAL were 26% more likely to have DOR than those in the bottom quartile [(PRAL (OR: 1.26; 95%CI: 1.08–1.42, *P* = 0.254)].

**Table 3:** Odds ratio (95% CI) of DOR according to quartiles of potential renal acid load (PRAL) and net endogenous acid production (NEAP)

	PRAL					NEAP				
	Q1	Q2	Q3	Q4	P-trend^a^	Q1	Q2	Q3	Q4	P-trend^a^
DOR/Control	(33/61)	(28/59)	(32/61)	(27/69)		(34/57)	(32/56)	(28/66)	(26/71)	
Crude	Ref (1.00)	1.22 (0.47–1.62)	1.24 (0.53-1.77)	1.28 (0.88–1.76)	0.380	Ref (1.00)	1.61 (0.33–2.13)	1.71 (0.38–2.31)	1.95 (0.52–2.75)	0.078
**Model 1**	Ref (1.00)	1.18 (0.64–1.63)	1.22 (0.72.-1.84)	1.26 (0.97–1.41)	0.258	Ref (1.00)	1.52 (0.27–2.01)	1.62 (0.32–1.20)	1.75 (0.39–2.44)	0.045
**Model 2**	Ref (1.00)	1.20 (0.64–1.62)	1. 24 (0.50.-1.91)	1.26 (1.08–1.42)	0.254	Ref (1.00)	1.49 (0.23–2.06)	1.68 (0.32–2.43)	1.72 (0.36–2.53)	0.075

Obtained from binary logistic regression by considering quartile of DAL (based PRAL and NEAP) as ordinal variable

Model 1: Adjusted for physical activity, energy intake

Model 2: Additionally, adjusted for fat mass and BMI

## Discussion

The present study is the first to examine the association between potential acid load of diet and risk of DOR in a case-control study. We found that diets with high acid-forming potential (reflected by high PRAL and NEAP scores) was associated with lower serum AMH levels and AFC in women with DOR. Also, high DAL based on PRAL score was positively associated with the risk of DOR in a full-adjusted model, as study participants in the highest PRAL score quartile had a nearly 1.4-fold higher risk of DOR in relation to women in the lowest quartile.

Ovarian reserve affects several aspects of reproductive health in women including female fecundity, fertility outcomes following infertility treatment, menopausal age and length of reproductive life-span [4]. Ovarian reserve gradually decreases with age, however the rate of its decline differs greatly among women in reproductive age, so factors other than age might influence ovarian reserve. In contrast to age and genetic factors, environmental factors affecting ovarian reserve can be modified, among which nutritional factors and dietary intakes have been focused recently to improve female follicular quantity and quality. Moslehi et al. systematically reviewed the current evidence on the association between nutritional factors, ovarian reserve markers and menopausal age and found serum 25- hydroxyvitamin D [25(OH)D] concentration and intake of soy products to potentially affect ovarian reserve [4].

The underlying mechanism of decreased ovarian reserve is complex and remains largely unknown. Several experimental studies have demonstrated the impact of oxidative stress and mitochondrial dysfunction on ovarian aging. The quantity and quality of oocytes are mainly determined by telomere length of granulosa cells, which is highly sensitive to the accumulation of intracellular reactive oxygen species (ROS) [12]. In this regard, antioxidant and scavenger compounds such as secoisolariciresinol diglucoside [12], resveratrol [21] and coenzyme Q10 [22, 23] improved ovarian reserve by inhibiting oxidative damage. As with ROS accumulation, low-grade inflammation is a hallmark of aging. limited evidence exists on the role of chronic low-grade inflammation in DOR. Liberos et al. found that inflammasome mediated low-grade inflammation contributes to decreased ovarian reserve in *Asc*^−/−^ and *Nlrp3*^−/−^ mice, highlighting that ovarian reserve could be improved by suppressing inflammatory pathways [24].

We found no previous studies examining the association between DAL and ovarian reserve; thus, it is challenging to interpret our findings based on existing literature. However, high DAL which results in low-grade metabolic acidosis has been associated with an increased risk of cardiovascular diseases and several cardiometabolic risk factors including insulin resistance, T2DM, obesity and dyperlipidemia in large population-based studies [7–10, 25]. In a study by Rezazadegan et al., high DAL was negatively associated with metabolic health status in overweight and obese adolescents, as adolescents in the highest tertile of PRAL and NEAP had higher odds of metabolically unhealthy overweight/obese status, based on International Diabetes Federation criteria, compared with those in the lowest tertile [26].

Women with DOR have been found to have increased risk for cardiovascular diseases [11]. It has been postulated that the decline in circulating AMH levels may be among the factors which mediate the increased cardiovascular risk in these women [27]. Based on recent experimental and observational studies, AMH, beyond its local role in ovarian follicle development, may be directly involved in cardiovascular physiology [27–29]. In this regard, an inverse association between AMH level and pregnancy-induced hypertension was found in several studies [30, 31]. AMH level was also associated with some metabolic risk factors such as insulin resistance and dyslipidemia in some [32, 33], but not all [34] observational studies conducted on the relationship between AMH and components of metabolic syndrome. In a study by Verit et al., cardiovascular risk markers including HOMA-IR, C-reactive protein (CRP), triglyceride and LDL cholesterol levels were significantly increased in women with DOR compared to women with normal ovarian reserve [35]. We found a negative association between DAL and serum AMH concentrations, suggesting the potential beneficial effect of lowering diet-induced acid load on reducing the cardiovascular risk in women with DOR.

Since, many physiological and cellular functions depend on acid-base equilibrium, chronic adherence to a diet with high potential acid load (defined by high consumption of animal products, processed foods and grains and limited consumption of most fruits and vegetables) may induce metabolic stress followed by chronic inflammation and metabolic disorders [36]. Reduction-oxidation reactions are among important pH dependent cellular reactions. Thus, dysregulation of the endogenous acid-base balance due to diet-induced metabolic acidosis favorites the accumulation of ROS, oxidative stress, inflammation and metabolic derangements by disrupting cellular physiological reactions. This may partially explain the positive association found in this study between higher acid-forming potential of diet and DOR.

Another possible mechanism which may explain the inverse association between DAL and ovarian reserve markers is the potential effect of diet-induced acidosis on increased adiposity. Several previous studies demonstrated the relationship between DAL and obesity measures [37, 38]. A 16-week randomized clinical trial on the effect of a plant-based diet with low DAL on body composition and insulin sensitivity on adults with overweight reported a greater reduction in body weight mainly due to a reduction in fat mass and visceral fat in participants on a low DAL diet compared to control group [39]. Several mechanisms were suggested in this regard. DAL-induced metabolic acidosis stimulates the secretion of glucocorticoids which results in the rise in fat mass and impairs insulin sensitivity [40]. Also, diet-induced acidosis has been proposed to increase renal magnesium loss, which may induce insulin resistance, and to decrease the level of adipokines resulting in increased appetite [41]. We have found that higher DAL is positively associated with FM among women with DOR. Since, serum AMH level is lower in overweight and obese women with DOR compared to nonobese women with DOR, high DAL may deteriorate ovarian reserve status by increasing the fat content of body in women with DOR [42].

The association between PRAL, NEAP and odds of decreased ovarian reserve was investigated in the present study for the first time. Large sample size and matching case and controls by age and BMI to reduce the effect of confounding variables were strengths of our study. However, several limitations of this study should be noted. As with all case-control studies, no causal association can be concluded between DAL and risk of DOR. Also, the effect of residual confounders such as mood status and genetic background should not be ignored which may affect our estimates. Although, we used a validated FFQ to estimate dietary intakes, measurement error and recall bias should be considered.

## Conclusion and future research

Diets with high acid-forming potential may be negatively associated with serum AMH levels and AFC in women with DOR. Also, diets with high PRAL may increase the risk of decreased ovarian reserve, suggesting that adherence to a low-DAL diet can improve the ovarian reserve among women with DOR. The association between DAL and the markers of ovarian reserve and cardiometabolic risk factors, both in women with DOR and women with normal ovarian reserve, should be examined in prospective studies and clinical trials. Also, future studies should be focused on exploring the mechanisms by which lowering acid load of diet can beneficially affect ovarian reserve.

## Data Availability

The datasets used and/or analyzed during the current study are available from the corresponding author on reasonable request.

## Declarations

### Attestation

Data regarding any of the subjects in the study has not been previously published unless specified. Data will be made available to the editors of the journal for review or query upon request.
